# Talks with Dr. Mandeville

**Published:** 1892-08

**Authors:** J. V. Mandeville


### HALL'S JOURNAL OF HEALTH

TRUTH DEMANDS NO SACRIFICE; ERROR CAN MAKE NONE.

Vol. 39. AUGUST, 1892. No. 8.

### TALKS WITH Dr. MANDEVILLE.

No. 7.

[Concluded.]

When we come up to the brain itself there is something more to be said. In the first place we have two knots which are difficult to distinguish in the spinal cord, perfectly distinct things, one is called the *optic thalamus*, being a latin word for "couch," the other is called the *corpus striatum*. These are two masses quite separate from each other, the sensory nerves go to the one, and the motor nerves, which go to the spinal cord, come from the other. The nerves which go to the spinal cord may be divided into those which go to the spinal cord and end there, and those which begin at the spinal cord and proceed from it; those which come from the brain down the spinal cord, and those which come back from the spinal cord to the brain. We are certain that there are some which go to the spinal cord and end there, and others which begin at the spinal cord and come away, because if the spinal cord is injured—if a person has a broken back—you can still produce convulsive motions of the legs by pricking them, or afflicting them in some other way. Messages will still be carried from the foot to the spinal cord when it is not possible to send them up to the brain. That which is called the "*reflex action*," was discovered by Dr. Marshall Hall; but there are others which run right up to the brain and come down again. The first office of the brain, therefore, is to receive messages in the *optic thalami*—to co-ordinate them there—to transmit them to the *corpora striata*, and tell them what to do with them.

But that is not all; messages, when they come up from all parts of the body into this portion of the gray matter, which is at the base of the brain, having been carried across, are sent to other parts of the body, and that is how you perform complicated actions without having your choice asked about them. If a cat comes suddenly round a corner and you jump out of the way, you perform an exceedingly complicated series of motions in consequence of the complicated sensations.

It is not like the case where a pressure upon the foot makes the foot get out of the way, but there is a complicated sense of vision and feeling, and from that you deduce an exceedingly complicated series of actions to get out of the way. There is a number of motions you have to perform—you have to keep yourself up to the balance and to get out of the way. You do that without being asked to do it, but as the result of a variety of complications.

In order to get a clearer notion of what is called the cerebral hemisphere—for it is in two parts, there being a furrow which divides them into two parts,—and "cerebral," meaning "brain,"—we must suppose that it is much thicker because it is doubled upon itself a great many times. That gray matter which is doubled upon itself to form the hemispheres occupying 300 square feet, which is an enormous area to think of as doubled up inside a man's skull, so that here we have a great piece of thin matter, that is to say, a great town of card-houses, any one of which is exceedingly sensitive and any one of which might be knocked down in a moment, and built up again by the blood. Each of these things is connected by white threads with the "streaked bodies" (the *corpora striata*) and the *optic thalamus*, that message goes up to the cerebral hemisphere; then the messages are interchanged by means of the white threads going across from one part of the cerebral hemisphere to the other (there is a great interchange of messages from all parts of the cerebral hemispheres), and then these messages come down from all parts of the "streaked body," and are sent out along the muscles.

When you have a choice, what you will do and what you will not do, there is a much more complicated thing which happens, that is to say, a message has gone up to the cerebral hemisphere, and has been dealt with by the sending of those messages along the nerves, and then messages sent along the "streaked bodies" to the nerves, telling them to perform the requisite motions. In the brain there is more than in the two knots of any part of the spinal cord. It is quite true that we have the two knots, but besides this structure we have the cerebral mass of gray matter which is put inside of your skull, and that may be consulted by one of the two knots before it sends on its message to the others. If the message is sent on straight—merely translated into shorthand and sent on—then you have a case which is just like automatic action of the spinal cords, except in the length of it. The spinal cord may act automatically, and you do not know it, because the message is sent up to one of those small brains in the spine, and has been sent down, so that you do not know it. If it has only gone to the small knot, and then been sent back again, you do a thing, but you have no choice about it. It is a thing which you do instinctively, without any choice, without thinking, "I shall be run over if I do not get out of the way." But if you have time and you consider, "Shall I be able to get across?" and then you come to the conclusion that you will be able to get across, there is a much longer interval which elapses between your sensation of the cab coming towards you and your determination of what you will do. You have to make a calculation, and that calculation is made in the brain by means of the white threads which go across, so that there are two distinct ways in which a connection may be established between the incoming message from the end of your body and the outgoing message which tells certain muscles to move. Now there is just one kind of message which goes in to the brain which I want to consider particularly, because it tells us more about the message from the outer world than any other, that is, the kind of message which comes in from the eye.

We are going to consider that in another paper, but I will just say in what way it is that the message comes in from the eye and is carried to the brain. There is in the back of the eye a skin of gray matter which is called the *retina*, which is connected with an innumerable number of nerve-fibres which go away into this great bundle of fibres, which is called the optic nerve, and the optic nerve goes down to the knot of gray matter called the optic ganglion, which is connected with the *optic thalami*.

Now the manner of transmitting the message is as follows: In front of the eye there is a lens, that is to say, a transparent body which is shaped like a burning glass, so that as the light falls upon it, it is made to converge to a point upon the other side. This light which falls upon the burning glass makes upon the skin a picture of the thing outside, which is just like a picture upon a ground glass of the photographer's camera, and when the light falls upon this sheet of gray matter, which is at the back of the eye, the picture disturbs it. The card-houses which are at the back of the eye are so exceedingly fragile that they fall down at once, and the nerves carry that message away into the brain, and that is one of the particular messages which is carried away from the brain, and which is acted upon by the brain in the form of sending that message by the nerves which move certain muscles.

Before concluding, I might say that I have chosen this subject in order to awaken an interest in the greatest possible number of other subjects. As I go on, we shall see that this particular subject we have been treating of is a sort of Clapham Junction of all the sciences in regard of the number of trains of thought which converge at this point, and which go out from it. In the first place we have a connection with physiology ; in the next place we have a connection with physics, as you will see on our next meeting, when we shall have to consider some of the properties of light ; and we have a connection with mechanics by means of the mechanical explanation of those actions which go on with us ; and we have a connection with a subject far more difficult than any of these, namely, the subject of consciousness—what it is that we see, whether we see rightly, and how it is we think. And also, it may be observed, as this is a sort of junction of all the lines of the sciences, that there are more trains of thought which go off the line just at this point than at any other.

I should like to recommend you to read, as bearing upon these subjects, Michael Foster's "Primer of Physiology." It does not go into any detail of the particular subject of which we have to treat, but it will teach you just as much physiology as is wanted before you *begin the study of the subject.*

My next subject will be "The Eye and Seeing."

J. V. Mandeville.

